# Cost-effectiveness analysis of psychosocial intervention for early stage schizophrenia in China: a randomized, one-year study

**DOI:** 10.1186/s12888-014-0212-0

**Published:** 2014-07-26

**Authors:** Zhanchou Zhang, Jinguo Zhai, Qinling Wei, Jingfeng Qi, Xiaofeng Guo, Jingping Zhao

**Affiliations:** Institute of Mental Health, the Second Xiangya Hospital, Central South University, Key Laboratory of Psychiatry and Mental Health of Hunan Province, National Technology Institute of Psychiatry, No. 139 Renmin Middle Road, Changsha, 410011 China; Jining Medical College, Jining, China; Department of Psychiatry, 3rd Affiliated Hospital of Sun Yat-sen University, Guangzhou, China

**Keywords:** Schizophrenia, Cost-effectiveness, Psychosocial intervention

## Abstract

**Background:**

A combination of psychosocial interventions and medications has been highly recommended as a successful treatment package for schizophrenia. Its cost-effectiveness has not been fully explored yet. The aim of the present analysis was to evaluate the cost-effectiveness of antipsychotics combined with psychosocial treatment and treatment as usual for patients with early-stage schizophrenia.

**Method:**

Patients with schizophrenia (N = 1, 268) were assigned to the combination of medication and psychosocial intervention or treatment as usual for up to 12 months. Cost analysis included direct medical costs, direct nonmedical costs and indirect costs. Quality-adjusted life year (QALY) ratings were assessed with Short- Form 6D.

**Results:**

Average monthly psychosocial intervention costs for combined treatment were higher than treatment as usual (p = 0.005), but no significant differences were found in direct costs, indirect costs, and total costs between two groups (all p-values ≥ 0.556). Combined treatment was associated with significant higher QALY ratings than treatment as usual (p = 0.039). Compared with treatment as usual, combined treatment resulted in a gain of 0.031 QALY ratings at an additional cost of US$ 56.4, yielding an incremental cost-effectiveness ratio of US$ 1819.4 per QALY gained.

**Conclusions:**

Despite some limitations, our results supported that medication combined with psychosocial treatment was more cost-effective than treatment as usual for patients with early-stage schizophrenia.

**Trial registration:**

clinicaltrials.gov Identifier: NCT00654576

**Electronic supplementary material:**

The online version of this article (doi:10.1186/s12888-014-0212-0) contains supplementary material, which is available to authorized users.

## Background

Schizophrenia is a severe and chronic illness as well as one of the most expensive illnesses to treat [1,2], which has a significant impact on individuals, families and societies in terms of both health and economic loss. Schizophrenia is also among the 15 leading causes of disability and the top 10 leading causes of years lost due to disability [3]. In China, there are about 10 million patients suffering from schizophrenia, and it is estimated that the annual economic cost of schizophrenia exceeds 1 billion RMB [4,5].

Although antipsychotic medications are the mainstay of the treatment for schizophrenia, patients with schizophrenia benefit more from the combined use of antipsychotic drugs and psychosocial treatment than pharmacotherapy alone in delaying or preventing relapse or reducing hospital days [7,8]. Symptomatic relapse or readmission in schizophrenia is both distressing and costly. It can devastate the lives not only of patients, but also of their families [9].

Most economic evidence in this area focuses on the cost-effectiveness of medications and different ways of organizing mental health care [10–12], and there is still little economic evidence on psychosocial intervention [13,14]. In a previous article, we reported a 1-year randomized clinical trial that examined the effect of medication combined with psychosocial intervention vs medication treatment alone on outcomes of patients with early stage schizophrenia [15].

The current article presents resulted on measures of health care costs and health related quality of life. The primary outcomes were total health costs and quality-adjusted life year (QALY) ratings. Based on a randomized controlled trial, the present study sought to examine whether medication combined psychosocial intervention represents a more cost-effective intervention than treatment as usual for patients with early stage schizophrenia.

## Methods

### Study design

The trial was a multicenter, randomized, and controlled study conducted between January 2005 and October 2007 at 10 clinical sites in China. The background, rationale, and methods of the Early-stage Schizophrenia Outcome Study (ESOS) have been presented in detail previously [15]. This study was approved by the institutional review board at *the Second Xiangya Hospital, Chongqing Mental Health Center, Psychiatric Hospital of Jiangxi Province, West China Hospital, Mental Hospital of Henan Province, Beijing Anding Hospital, Shanghai Mental Health Center, Guangzhou Brain Hospital, Hunan Brain Hospital, and Nanjing Brain Hospital.* Written informed consent was obtained from the patients or their legal guardians. Additional files 1 and 2 comprise the the full trial protocol and consort statement extension for cluster randomized controlled trials respectively.

Participants were randomly assigned to receive the combination of medication and psychosocial treatment or treatment as usual (medication treatment and brief intervention) and were monitored for up to 12 months or until medication treatment was discontinued for any reason after baseline assessment. Group assignment was based on a 1:1 randomization scheme balanced by sites and medication prescribed by independent investigator according to a computer generated randomization list.

### Participants

Study participants were enrolled from outpatient psychiatric clinics and under maintenance treatment. Inclusion criteria in this study were: (1) age 18 to 50 years old; (2) met DSM-IV diagnostic criteria for schizophrenia or schizophreniform disorder, as determined by the Structured Clinical Interview for DSM-IV Axis I Disorders–Clinician Version administered by study investigators[16]; (3) length of illness ≤ 5 years; (4) stable clinical condition (Positive and Negative Syndrome Scale PANSS, [17] total scores ≤60); (5) treated with one of the following 7 oral antipsychotics: chlorpromazine, sulpiride, clozapine, risperidone, olanzapine, quetiapine or aripiprazole. Patients were excluded if they were: (1) prescribed two or more antipsychotics or long-acting injectable antipsychotics; (2) participating in other therapy programs; (3) pregnant or breastfeeding; or (4) having serious and unstable medical condition.

### Intervention

All patients continued taking the original antipsychotic during the 12-month follow-up period. If a patient’s medication was stopped or switched, patients were classified as discontinued and terminated from the study.

The psychosocial intervention was reported in detail elsewhere [15]. In brief, patients who were assigned to the combined treatment group received medication treatment and were enrolled in a psychosocial intervention program. The psychosocial intervention included 4 evidence-based practices: psychoeducation, family intervention, skills training, and cognitive behavior therapy was conducted monthly for 12 months. Participants received the 4 interventions on the same day, for a total of 48 one-hour sessions. A lunch break and 2 half-hour breaks were provided to maintain engagement and attention. Therapists who had at least 2 years of clinical experience after earning an MD or PhD or at least 5 years of experience after earning a masters degree in clinical psychology delivered the psychosocial intervention.

The brief intervention included case management with antipsychotic medication and supportive interventions, which were provided monthly for 12 months.

### Measuring service use and costs

Costs estimates included direct medical costs, direct nonmedical costs, and indirect nonmedical costs. Direct medical costs consisted of intervention costs such as medication tests, therapy and so on, and uptake of health care services, including costs of medication. Service use was documented through a questionnaire completed by the caregiver at the end of every month during the study. Costs were calculated in US dollar ($) for the reference year 2005 in China.

The costs of psychosocial interventions consisted of start-up costs (development and training) and ongoing costs (services, supplies, travel and salary). We assumed that the psychosocial intervention protocol would be prepared by 2 specialists for 120 hours each and a training program would be conducted for 40 clinicians from all 10 psychiatric hospitals. The intervention program would consist of 42-hour sessions by two therapists [15]. We assumed that each session would have 12 participants (6 patients and 6 family carers). The unit cost for each medical procedure was calculated from the price system database built by the National Development and Reform Commission of China [18]. Direct nonmedical costs consisted of costs for traveling and parking. These costs were valued at US $ 0.1 per kilometer and US $ 0.5 per hour parking time.

Indirect nonmedical costs arise when production losses occur due to illness. Production losses can occur under 3 conditions. Patients were absent from paid work due to sick leave (work loss days), or they were ill but continue to work with reduced efficiency (work cutback days), or caregivers were absent from paid work due to take care of patients.

Cost-effectiveness analysis requires a single measure of health related quality of life that reflects both health gains and health losses. Participants were asked to complete Medical Outcome Study Short-Form 36-item questionnaire (SF-36) [19] at baseline, 6 months, and 12 months. The utility scores of the SF-6D derived from responses to eleven questions on the SF-36 questionnaire are used to calculate the QALYs gained during the follow-up period by weighing the length of time spent in a particular health condition by the utility. The SF-6D has 6 dimensions (physical functioning, role limitations, social functioning, pain, mental health and vitality) which have from 4 to 6 levels of severity [20]. The SF-6D scoring algorithm has been validated and established for the adult Chinese population in Hong Kong in previous studies [21,22]. The general population mean SF-6D preference value is 0.787, which was estimated from the SF-36 data of a general population survey of 2410 adult Chinese in Hong Kong in 1998 [23].

Other outcomes further assessed treatment effectiveness by measuring rates of treatment discontinuation, symptom severity (PANSS) [17] and global severity of illness (Clinical Global Impressions Scale) [24]. The social functioning and family burden were rated by the Global Assessment Scale (GAS) [25] and Family Burden Scale (FBS) [26], respectively. Agreement among the raters was high for the PANSS, CGI and GAS (Pearson correlation coefficient, 0.78-0.86) at baseline and every 6 months. All raters were kept blind for patient's treatment condition.

### Statistical analysis

All analyses were conducted with the Statistical Package for Social Sciences, version 15.0 (SPSS Inc, Chicago, Illinois). Baseline characteristics were compared between the two groups by analysis of variance, Pearson’s chi-square test, or Fisher’s exact test, as appropriate. Average monthly costs were compared between the groups with use of a mixed model including the same fixed covariates as for the time to discontinuation, plus baseline value, time, the interaction between treatment and time. Time was classified into months (baseline, 3, 6, 9, and 12 months). All statistical tests were two-tailed.

In the cost-effectiveness analysis, the average benefit in terms of Quality adjusted life year (QALY) gained and the average cost was calculated for each treatment. The average cost-effectiveness ratio (CER) of each treatment was obtained. We measured the incremental cost-effectiveness ratio (ICER), defined as extra cost per QALY gained [14,27] with the combined medication and psychosocial intervention (Combined treatment) and treatment as usual (TAU).

$$ \mathrm{ICER} = \left({\mathrm{Cost}}_{\mathrm{Combined}\ \mathrm{treatment}}-{\mathrm{Cost}}_{\mathrm{TAU}}\right)/\left({\mathrm{Effect}}_{\mathrm{Combined}\ \mathrm{treatment}}-{\mathrm{Effect}}_{\mathrm{TAU}}\right) $$

A strategy was considered as cost-effective versus treatment as usual when ICERs were below the cost-utility threshold acceptable in China. We used 3 × the per capita GDP of China in 2005 (US $ 5,100)/QALY as the threshold according to WHO recommendation [28,29].

### Sensitivity analysis

In this study, one-way deterministic analysis was carried out to test the model’s robustness. One-way deterministic analysis included modifications of the following inputs: the number of subjects per workshop that was varied from 5 to 3, and the clinical effect and the cost of per workshop that were varied by ± 20%. The psychosocial intervention workshops were designed to be delivered to 6 patients, and the total cost of providing the intervention is the same even if fewer subjects attend. The per-person cost of the intervention therefore varies depending on the number of subjects per workshop. Assuming that the effectiveness of the intervention was the same, we decreased the number of subjects per workshop in sensitivity analysis.

## Results

### Participants

**Figure 1:**
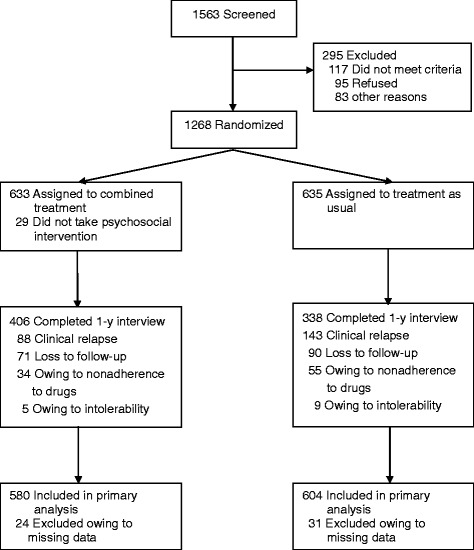
**Flowchart of participation in the study.**

Altogether, 1268 participants entered the study, 633 were assigned to receive antipsychotics combined with psychosocial intervention (of whom 29 refused the psychosocial intervention) and 635 to receive treatment as usual. Baseline utilization data were not available for 55, leaving 1184 patients for analysis (Figure 1).

There were no significant differences between study groups with respect to baseline demographic and clinical characteristics (all p-values ≥ 0.057). The mean age was 26 years, and 55 percent of the patients were male (Table 1).

**Table 1:** **Patient characteristics at the time of random assignment by initial treatment**

**Characters**	**Combined treatment (n = 580)**	**Treatment as usual (n = 604)**	**F/** ***χ*** ^**2**^	**p**
Age, y	26.1(7.6)	26.3(8.0)	0.237	0.626
Male No.(%)	312(53.8)	336(55.6)	0.403	0.526
Marital status No.(%)			3.113	0.211
Married	143(24.7)	164(27.2)		
Previously married	37(6.4)	26(4.3)		
Never married	400(69.0)	414(68.5)		
Education, y	12.2(2.9)	11.9(2.9)	2.564	0.110
Duration of schizophrenia, mo	22.5(20.0)	24.7(21.0)	3.462	0.063
PANSS total score	44.6(13.5)	45.3(13.9)	0.799	0.372
CGI severity score	2.5(1.2)	2.6(1.2)	0.849	0.357
GAS total score	74.2(11.8)	74.2(11.9)	0.000	0.984
FBS total score	12.3(7.6)	12.6(7.9)	0.468	0.494
SF-36				
PCS	49.6(7.4)	51.0(16.6)	3.621	0.057
MCS	50.3(10.5)	49.4(12.5)	1.617	0.204

PANSS = Positive and Negative Syndrome Scale; CGI = Clinical Global Impressions Scale; GAS = Global Assessment Scale; FBS = family burden scale of diseases; SF-36 = the 36-Item Short Form Health Survey; PCS = the Physical Component Score; MCS = the Mental Component Score.

In the intention-to-treat analysis using all available follow-up data, 62.8% of patients completed 1-year evaluation, with significant differences in the proportion of participants in two treatments groups (70.0% for combined treatment group and 56.0% for treatment as usual group, *χ*^2^ = 24.975, df = 1, p < 0.001).

### Costs

Average monthly costs by treatment groups were showed in Table 2. The majority costs were attributable to the direct costs. Average monthly psychosocial intervention costs were higher for combined treatment than treatment as usual (p = 0.005), but no significant differences were found in direct costs, indirect costs, and total costs between two groups (all p-values ≥ 0.556).

**Table 2:** **Average monthly costs by treatment group over 12 months**

**Type of cost (US $)**	**Combined treatment (n = 580)**	**Treatment as usual (n = 604)**	**F**	**p**
Direct medical costs	112.2(8.9)	107.5(9.2)	0.167	0.918
Medication costs	77.7(7.9)	79.2(8.1)	0.014	0.998
Intervention costs	6.9(1.7)	1.8(1.3)	7.837	0.005
Health care use costs^a^	25.7(3.9)	25.4(3.8)	0.141	0.707
Direct nonmedical costs	7.4(1.3)	6.5(1.4)	0.226	0.878
Indirect costs	94.7(14.0)	97.1(14.4)	0.007	0.999
Total costs	213.3(18.6)	213.2(19.2)	0.116	0.951

^a^Excluded intervention costs.

### Effectiveness

**Figure 2:**
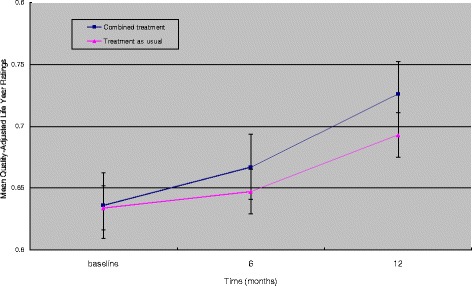
**Average monthly quality-adjusted life year rating.**

As shown in Figure 2, significant improvement in QALY ratings was found from baseline to 12 months. Combined treatment was associated with higher QALY ratings than treatment as usual (p = 0.039). Table 3 reported mixed model-adjusted means of the effectiveness measured by treatment groups across months 6 and 12. Differences between two treatment groups were also observed in FBS total scores and SF-36 the Physical Component Score (PCS) and the Mental Component Score (MCS) scores (all p-values ≤ 0.042).

**Table 3:** **Comparison of effectiveness by Treatment Groups (adjusted means across all points based on mixed models)**

**Effectiveness measure**	**Combined treatment (n = 580)**	**Treatment as usual (n = 604)**	**F**	**p**
QALYs	0.676(0.004)	0.658(0.004)	3.259	0.039
SF-36				
PCS	50.6(0.3)	50.3(0.2)	4.650	0.010
MCS	51.5(0.3)	49.1(0.3)	3.643	0.026
FBS	8.6(0.2)	10.1(0.2)	2.484	0.042
PANSS total scores	38.5(0.2)	40.0(0.2)	0.406	0.810

QALYs = quality-adjusted life-years; PANSS = Positive and Negative Syndrome Scale; SF-36 = the 36-Item Short Form Health Survey; PCS = the Physical Component Score; MCS = the Mental Component Score; FBS = family burden scale of diseases.

Compared with treatment as usual, combined treatment resulted in a gain of 0.031 QALYs at an additional cost of US$ 56.4, yielding an incremental cost-effectiveness ratio of US$ 1819.4 per QALY gained. Considering the common threshold accepted in China (US$ 5,100 per QALY gained), combined treatment could be considered a cost-effective option compared to treatment as usual.

At the end of the study, 14.6 percent of patients in the combined treatment group and 22.5 percent of patients in treatment as usual group had relapsed (*χ*^2^ = 12.899, df = 1, p < 0.001); readmission occurred in 6.5% of patients in the combined treatment group and 11.2% of patients in treatment as usual group (*χ*^2^ = 8.540, df = 1, p = 0.003).

### Sensitivity analyses

Table 4 showed the details of the sensitivity analysis performed. The combined treatment was always a dominant strategy over treatment as usual.

**Table 4:** **Sensitivity analysis**

**Parameter**	**Average CER (US $/QALY)**	**ICER**
Base case (BC)	14960	1819.4
Patients attendance per workshop		
5 cases (−16% BC)	15144	2354.8
4 cases (−33% BC)	15422	3161.3
3 cases (−50% BC)	15882	4496.8
Cost per workshop		
+20% BC	15093	2206.5
- 20% BC	14827	1432.3
Clinical effect		
+20% BC	12467	1151.0
- 20% BC	18700	4338.5

QALYs = quality-adjusted life-years; CER = incremental cost effectiveness ratio; ICER = incremental cost effectiveness ratio.

## Discussion

Schizophrenia is a very serious costly mental illness [1,2]. In this relatively large clinical trial of medication combined psychosocial intervention for outpatients with early-stage schizophrenia, we found that combined treatment group had slightly higher QALYs than treatment as usual group, but there were no statistically significant differences in medication costs or total health costs. These results were consistent with previous reports [27,28] in which patients were treated with medication and psychosocial intervention substantially increased health gain with lower medical cost. The differences between two groups in FBS total scores and SF-36 PCS and MCS scores in present study further supports previous studies [6,7,27].

These results extend the efficacy and safety outcomes analysis from the first report of the ESOS study, which showed that patients with early-stage schizophrenia receiving combined medication and psychosocial intervention had a lower rate of treatment discontinuation or change, a lower risk of relapse, and improved insight, quality of life, and social functioning compared with those receiving treatment as usual [15]. Average monthly psychosocial intervention costs were higher for combined treatment than treatment as usual, but no significant differences were found in direct costs, indirect costs, and total costs Individual payments play a huge role in the current Chinese health care system. Patients with schizophrenia have to stay at home to take medicine except for hospitalization in mental hospital or seeing doctor in out-patient in China [29].

Family caregivers experience significant burden in taking care of their patients with schizophrenia, and family members may influence their relative's adherence with medication and other treatment regimens [30]. In our study, the family caregivers of patients assigned to combined treatment reported less burden overall over 12 months than the caregivers of patients assigned to medication only treatment, suggesting that combined treatment may decrease the family burden.

This study has several limitations. First, this was a 12-month trial; a longer-term randomized clinical trial would contribute substantially to understanding cost-effectiveness of psychosocial intervention on schizophrenia. Second, data loss from attrition was considerable. More patients in medication group dropped out for medication discontinuation or relapse than combined treatment group. Partial compliance with antipsychotic medication is associated with an increased risk of inpatient hospitalization. As we all know, relapse and rehospitalization contribute significantly to the economic burden of schizophrenia [9]. However, we did not continue to collect any information when medication discontinuation or clinical relapse happened, also did not measure costs associated with relapse. This may be the important reason why combined psychosocial intervention was substantially more effective (using rates of discontinuation for any cause as the measure of effectiveness), but slightly higher QALYs than medication treatment alone in present study. Third, we used self-report for most of the measures. Self-report can be vulnerable to recall bias. The self-report of medication and care products for example, is often underestimated [31]. Last, this study recruited only patients with early-stage schizophrenia. Further prospective data monitoring studies are needed to evaluate chronic schizophrenic patient costs.

## Conclusion

A combination of psychosocial interventions and medications has been highly recommended as a successful treatment package for schizophrenia [6]. Despite there were some limitations, present results confirmed that combined intervention package was more cost-effective than treatment as usual in early stage schizophrenia. Future research is needed to evaluate the longer-term effects on patient outcomes and consider broader measures of health care resource utilization.

## Additional files

Additional file 1:
**Protocol.** Antipsychotic combination with psychosocial intervention on the early-stage schizopherenia outcome study. Additional file 2:
**The consort statement for cluster randomised controlled trials.**
