# Supplementary material for: Cost-effectiveness analysis of psychosocial intervention for early stage schizophrenia in China: a randomized, one-year study
Source: BMC Psychiatry. 2014 Jul 26;14:212. doi: 10.1186/s12888-014-0212-0 (PMC4149262; doi:10.1186/s12888-014-0212-0)
Supplement: Additional file 1: — Protocol. Antipsychotic combination with psychosocial intervention on the early-stage schizopherenia outcome study. [file 12888_2014_212_MOESM1_ESM.pdf]

Protocol

Antipsychotic Combination with Psychosocial Intervention on the Early-stage Schizophrenia Outcome Study

PI: Jingping Zhao & Xiaofeng Guo

Synopsis

Antipsychotic Combination with Psychosocial Intervention on the Early-stage Schizophrenia Outcome Study

Introduction

Long-term treatment effectiveness has remained a major problem for schizophrenic patients. Pharmacotherapy alone is insufficient to prevent relapses or to ensure recovery of functional disabilities in the illness course. In an attempt to improve the long-term outcome for individuals with schizophrenia, there is growing interest in psychosocial interventions as an adjunct to facilitate recovery from an initial episode of psychosis and reducing the long-term disability resulted from schizophrenia.

Objectives

The primary objective is to evaluate rate of treatment discontinuation and time to treatment discontinuation comparing combined treatment and medication treatment alone.

The secondary objectives are to assess: changes of psychopathology in schizophrenia, side effects, subjective attitudes toward medication, social function and cognitive function, quality of life, family/carer burden, and cost-effective analysis.

Study design

The study is a multicenter, randomized and controlled study with an intervention period of 1 year and assessments of participants at baseline, 3, 6 months and every 3 months thereafter. An additional visit will be paid in case of discontinuing and/or changing medication, hospitalization and/or relapse (for definitions see below). The 10 clinical sites is *the Second Xiangya Hospital, Chongqing Mental Health Center, Psychiatric Hospital of Jiangxi Province, West China Hospital, Mental Hospital of Henan Province, Beijing*

Anding Hospital, Shanghai Mental Health Center, Guangzhou Brain Hospital, Hunan Brain Hospital, and Nanjing Brain Hospital in China.

Participants will be randomly assigned to receive the combination of medication and psychosocial treatment or medication alone and are monitored for up to 12 months or until medication treatment is discontinued for any reason after baseline assessment. Group assignment is based on a 1:1 randomization scheme balanced by sites and medication prescribed by independent investigator according to a computer generated randomization list.

Study population

The study will enroll approximately 1,300-1400 subjects with schizophrenia, who are randomly distributed to receive antipsychotic drugs combined with psychosocial intervention or antipsychotic drugs alone based. The sample sizes are selected to make possible the detection of a 15 percent difference in discontinuation rates after one year with 85 percent power and a two-tailed alpha level of significance of 0.05.

Inclusion criteria

All patients are enrolled from outpatient psychiatric clinics and under maintenance treatment. Eligible patients are 16 to 50 years of age who have to meet following enrollment criteria:

- DSM-IV diagnosis of schizophrenia or schizophreniform disorder within the past five years, as determined by the Structured Clinical Interview of the Diagnosis (SCID) administered by study investigators or trained staff;
- living with family members who could be involved in the patient's care;
- PANSS (Positive and Negative Syndrome Scale) total scores are ≤60;
- on maintenance treatment with one of the following 7 oral antipsychotics: chlorpromazine, sulpiride, clozapine, risperidone, olanzapine, quetiapine or aripiprazole.

We select these 7 antipsychotics because over 90% of schizophrenia patients in China are prescribed one of these antipsychotics.

Exclusion criteria

- Patients are excluded if they are:
- prescribed two or more antipsychotics or long-acting injectable antipsychotics;
- participating in other therapy programs;
- pregnant or breastfeeding;
- a serious and unstable medical condition.

Treatment

Pharmacotherapy

Because all patients are on maintenance treatment, we encourage clinicians to try to keep patients on the same medication for at least 3-6 months in order to gauge treatment efficacy and minimize early discontinuation. However, medications could be changed at any time during the course of the study if the change is clinically warranted. If a patient’s medication is stopped or switched, patients are classified as discontinued and terminated from the study. No further assessments will be required for these patients.

Concomitant medication

Mood stabilizers, benzodiazepines, antidepressants, and anticholinergic medications are permitted, and daily doses of all medications are recorded throughout the study.

Brief intervention

In medication alone group, the brief intervention will also be given to paitents, which included case management with antipsychotic medication and supportive interventions, which are provided monthly for 12 months.

Psychosocial intervention

Patients will be assigned to the combined treatment group received

medication treatment and are enrolled in a psychosocial intervention program. The psychosocial intervention strictly followed a detailed treatment manual designed by the principal investigators and included four evidence-based practices: psycho-education, family intervention, skills training and cognitive-behavioral therapy. Psychosocial intervention participants are seen 12 times (once per month for 12 months), receiving each of the four group treatments on the same day, for a total of 48 one-hour sessions (see Table 1 for topics covered). A lunch break and two half-hour breaks are provided to maintain engagement and attention. We have designed this comprehensive psychosocial intervention to be delivered on the same day once a month mainly due to the care structure in China, the potential time and cost burden to patients and their family members, as well as feasibility of being adopted by other care settings. In China, the vast majority of schizophrenia patients live with their family members because of limited social welfare for severe mentally ill patients. Many of these family members also work full time so it is not convenient for them to take time off every week and bring the patients for therapy. In addition, all of our psychosocial interventions are group based, so having many patients and their family members come in once a week at the same time is not feasible and practical. Weekly intervention visits also would have increased the costs of transportation and therapist time, making the overall cost of the psychosocial intervention higher. Finally, psychosocial interventions have become more popular in recent decades in China, but the number of well-trained therapists remains limited in many Chinese psychiatric settings. More frequent therapy sessions could not only be difficult for patients and family members, but also hard to adopt by many psychiatric settings.

Psycho-education included teaching patients and caregivers about the symptoms, treatment and course of mental illness, and afforded patients and family members the opportunity to ask questions about psychiatric disorders and treatment options. This group provided a forum in which to discuss

concerns and obtain support from the group in order to reduce the stigma of mental illness. The purpose of psycho-education is to increase patients' and caregivers' knowledge and understanding of the illness and treatment.

Family intervention included developing collaboration with the family; socializing about non-illness-related topics; monthly updates on each family's situation; enhancing family communication; teaching patients and their families to cope with stressful situations and the illness; and teaching patients and their families to detect signs of relapse and intervene in crises.

Skills training included modules on medication management and symptom self-management, dealing with stigma, social problem solving and independent living skills. The training included teaching complex interpersonal skills by breaking down the targeted behaviors into component steps and systematically using modeling, behavioral rehearsal, positive and corrective feedback, and in-vivo practice to shape the acquisition and generalization of skills.

Cognitive-behavioral therapy involves treatment of auditory hallucinations and delusions, associated symptoms and problems (i.e. anxiety, depression, and self-esteem), relapse prevention, and enhancing medication adherence. Treatment included an assessment and engagement phase, education, and building a therapeutic alliance; functional analysis of key symptoms, leading to formulation of a problem list; development of a normalizing rationale for the patients' psychotic experiences; exploration and enhancement of coping strategies; and addressed concomitant affective symptoms using relaxation training.

Therapists who have at least two years of clinical experience after earning an M.D. or Ph.D. or at least five years' experience after earning a masters degree in clinical psychology delivered the psychosocial intervention. They attend training workshops until they had mastered all treatment procedures. Treatment fidelity is maintained by having the therapists' supervisors assess adherence to the treatment manual after each monthly session by reviewing

videotapes.

Outcome measures

All subjects are assessed monthly by the study psychiatrists and every 2 weeks by a research assistant who had instructions to contact the psychiatrist if medication discontinuation, relapse or other problems are suspected. The psychiatrists assess patients mainly for medication management purposes, evaluating for clinical response to medications, medication compliance, and major side effects. The research assistants assess patients, patients' caregivers, and other sources every two weeks by phone for any hospitalizations, relapses, or other causes of treatment discontinuation. The research assistants also administer the symptom and functioning rating scales at scheduled intervals.

The primary measure is rate of treatment discontinuation or change and time to treatment discontinuation. Once a patient discontinued the study, no further assessments are completed. Treatment discontinuation criteria:

- clinical relapse/hospital admission;
- lost to follow-up or patient's refusal;
- noncompliance, defined as taking less than 70% of prescribed medications, detected either by the treating psychiatrist or research assistants during follow-up assessments;
- changing or stopping of initial antipsychotic by doctor or patient request
- intolerability, defined as severe side effects that caused the treating psychiatrists to stop the medications.

Clinical relapse is defined by any one of the following:

- psychiatric hospitalization;
- an increase in the level of psychiatric care (e.g., from clinic visits to day treatment) and a 25% or more increase in the PANSS total score (or 10 points if the initial score is 40 or less);

- a Clinical Global Impressions (CGI) Scale score of “much worse” or “very much worse”;
- deliberate self-injury;
- emergence of clinically significant suicidal or homicidal ideation;
- violent behavior resulting in significant injury to another person or significant property damage.

Secondary outcomes are different dimensions of psychopathology in schizophrenia, side effects, subjective attitudes toward medication, social function and neurocognitive function, quality of life, family/carer burden, and cost-effective analysis.

The following instruments are selected to measure the above mentioned outcomes:

- The Positive and Negative Syndrome Scale (PANSS) and Montgomery and Asberg depression scale (MADS) are used to assess psychopathological symptoms.
- The severity of psychopathology is rated by Clinical Global Impression (CGI).
- The Insight and Treatment Attitudes Questionnaire (ITAQ) is used to measure awareness of illness and insight into need for treatment in patient with schizophrenia.
- Quality of life is assessed with the Medical Outcomes Study 36-Item Short Form Health Survey (SF-36), which consists of 8 domains that assess bodily pain, general health, mental health, physical functioning, role emotional, role-physical, social functioning, and vitality.
- Social function outcomes are assessed using the Global Assessment Scale (GAS), Social Disability Screening Schedule (SDSS) and the Activities of Daily Living Scale (ADL).
- Cognitive Assessments. The cognitive battery included following five cognitive tests:

- *Wechsler Memory Scale-Revised Visual Reproduction Test* (Wechsler, 1987). This is a test of memory for nonverbal stimuli measuring visuomotor speed. For this protocol, the outcome measure is immediate delayed memory raw scores.
- *Wechsler Adult Intelligence Scale-Revised Digit Symbol Test* (Wechsler, 1981). This is a test of psychomotor speed and attention, in which the participant is allowed 120 seconds to move through a grid of 133 numbers (1 to 9) and pair them with the correct symbol by using a key consisting of nine symbol-digit pairs. The outcome measure is the number of correct symbols drawn within 120 seconds.
- *Computerized Wisconsin Card Sorting Test* (128-card version) (Heaton et al.,1993). This is a commonly used test of executive functioning, measuring cognitive flexibility, maintenance of a cognitive set, and working memory. In this test, the outcome measures are the number of categories completed and the number of perseverative errors made.
- *Trail Making Tests, Part A and B* (Reitan and Wolfson.,1993). In Part A, subjects draw lines connecting circles that are numbered. In Part B, subjects connect circles by alternating between ascending numbers and letters in alphabetical order. The outcome measures are the time to complete the tests.
- *Wechsler Adult Intelligence Scale-Revised Digit-span Task Test* (Wechsler,1981). Subjects are given sets of digits to repeat initially forwards then backwards. The number of correctly recalled trials is counted for forward and backward span.

- Costs estimates. Costs included direct medical costs, direct nonmedical costs, and indirect nonmedical costs. Direct medical costs consisted of intervention costs such as medication tests, therapy and so on, and uptake of health care services, including

costs of medication. Service use is documented through a questionnaire completed by the caregiver at the end of every month during the study. The costs of psychosocial interventions consisted of start-up costs (development and training) and ongoing costs (services, supplies, travel and salary). Direct nonmedical costs consisted of costs for traveling and parking. Indirect nonmedical costs arise when production losses occur due to illness.

- Family burden are rated by Family Burden Scale (FBS).
- Safety measures. The physical examination is recorded regularly. The Treatment Emergent Symptom Scale is used for monitoring adverse effects.

Statistical Methods and Analytic Plan

Analyses will be performed according to the intention-to-treat principle. Randomized patients who have at least one assessment during treatment made up the intention-to-treat population. Descriptive statistics will be provided for all data broken down by treatment group, by visit and by site. Continuous variables will be described by mean, standard deviation, range and number of observations. Discrete variables will be described by frequencies and percentages. All statistical tests will be carried out as two tailed tests; and alpha (level of significance) is set at 5%. Differences in continuous variables will be tested with analysis of variance, with treatment sites included as covariates. Discrete variables are analyzed with Chi-Square or Fisher’s Exact Tests. The primary objective of this study will be examined with survival analysis, both Kaplan Meyer Survival Analysis and log-rank test. Repeated measurements analysis will be performed, where applicable, for analysis of the secondary objectives.

Quality control

There are 10 sites participate which are led by the Second Xiangya

Hospital of the Central South University, who is the trial originator. The participating sites are Beijing Anding Hospital, Nanjing Brain Hospital, Shanghai Mental Health Centre, Jiangxi Province Mental Health Hospital, Guangzhou Brain Hospital, Chongqing Mental Health Centre, Henan Province Mental Health Hospital, Huaxi Hosptial of Sichuan University and Hunan Brain Hospital.

The Second Xiangya Hospital of the Central South University coordinates monitoring and data entry. A study start-up meeting is an efficient strategy to initially familiarized site staff with the protocol and provided study procedure training. The trial originator has provided instructional materials to all study sites and organizes an initial five-day training event that occurred prior to the implement of the trial. Reliability assessment of the major rating scales has been carried out. The originators of the intervention provide initial training in the procedure and monthly supervision of videotaped sessions.

Table 1. Content of monthly psychosocial treatment sessions

	Psycho-education Topics	Family Intervention Topics	Skills Training Topics	Cognitive Behavioral Therapy Topics
Month 1	Introduction into the program Discussion of goals and questions	Introduction into the program Discussion of goals and questions	Medication management(1)Identifying benefits of antipsychotic medication	Develop the therapeutic alliance
Month 2	What is schizophrenia?	The role of the family in schizophrenia	Medication management(2) self-administration and evaluation of medication	Using the 'ABC' Model to find connections between Activating Events, Beliefs, and Consequences.
Month 3	Causal and triggering factors	Relatives shared the experiences of caring for patients	Medication management(3) side effects of antipsychotic medication	Intervening with Auditory Hallucinations (Voices)
Month 4	A description of the various symptoms	Coping Strategies. Identify, describe, clarify, and teach coping strategies that are used by families.	Symptom management (1)identifying warning signs of relapse	Intervening with Auditory Hallucinations (Voices)
Month 5	Patients' concepts of illness and the Vulnerability-stress-coping-model	Coping Strategies. Identify, describe, clarify, and teach coping strategies that are used by families.	Symptom management (2) developing a relapse prevention plan	Intervening with Delusions (1)
Month 6	Course and outcome	Help families with problem-solving	Verbal and nonverbal communication (1)	Intervening with Delusions (2)
Month 7	Treatment recommendations concerning pharmacotherapy	Help families with problem-solving.	Verbal and nonverbal communication (2)	Intervening with anxiety, depression, and self-esteem
Month 8	Risks associated with treatment withdrawal	Family communication	Learn and practice problem solving skills	Intervening with anxiety, depression, and self-esteem
Month 9	Early detection of relapse	Family communication	Learn and practice problem solving skills	Relapse prevention
Month 10	Pregnancy and genetic counseling	Behavior management	Job-finding skills	Relapse prevention
Month 11	Talking over of open questions	Behavior management	Independent living skills	Enhancing medication adherence
Month 12	Final session – review of content	Final session – review of content	Independent living skills	Enhancing medication adherence

Table 2 Time and events schedule

	Screen/ Run-In	Follow-up				Early withdrawal visit
**Visit**	1	2	3	4	5	
**Month**	0	3	6	9	12	
**Informed consent**	X					
**Demographic and medical recording**	X					
**Vital signs**	X	X	X	X	X	X
**Physical exam**	X	X	X	X	X	X
**Clinical lab tests**	X	X	X	X	X	X
**Electrocardiogram**	X	X	X	X	X	X
**Psychiatric evaluation**	X	X	X	X	X	X
**Inclusion/exclusion criteria**	X					
**Randomization**	X					
**PANSS**	X	X	X	X	X	X
**MADS**	X	X	X	X	X	X
**CGI**	X	X	X	X	X	X
**ITAQ**	X	X	X	X	X	X
**SF-36**	X		X		X	X
**GAS**	X		X		X	X
**SDSS**	X		X		X	X
**ADL**	X		X		X	X
**Cognitive battery**	X		X		X	X
**FBS**	X	X	X	X	X	X
**TESS**	X	X	X	X	X	X
**Cost recording**		X	X	X	X	X
**Adverse event monitoring**	X	X	X	X	X	X
**Medication treament review**	X	X	X	X	X	X
**Psychosocial intervention**		X	X	X	X	X
**Concmitant medication review**	X	X	X	X	X	X
**Treatment discontinuation evaluation**						X

All subjects are assessed monthly by the study psychiatrists and every 2 weeks by a research assistant who had instructions to contact the psychiatrist if medication discontinuation, relapse or other problems are suspected.
